# Randomized, sham-controlled, clinical trial of repetitive transcranial magnetic stimulation for patients with Alzheimer’s dementia in Japan

**DOI:** 10.3389/fnagi.2022.993306

**Published:** 2022-10-13

**Authors:** Youichi Saitoh, Koichi Hosomi, Tomoo Mano, Yasushi Takeya, Shinji Tagami, Nobuhiko Mori, Akiyoshi Matsugi, Yasutomo Jono, Hideaki Harada, Tomomi Yamada, Akimitsu Miyake

**Affiliations:** ^1^Department of Neuromodulation and Neurosurgery, Osaka University Graduate School of Medicine, Osaka, Japan; ^2^Department of Geriatric and General Medicine, Osaka University Graduate School of Medicine, Osaka, Japan; ^3^Department of Psychiatry, Osaka University Graduate School of Medicine, Osaka, Japan; ^4^Faculty of Rehabilitation, Shijonawate Gakuen University, Osaka, Japan; ^5^Faculty of Health Sciences, Naragakuen University, Nara, Japan; ^6^Department of Medical Innovation, Osaka University Hospital, Osaka, Japan

**Keywords:** MMSE, MoCA-J, ADAS-cog, repetitive transcranial magnetic stimulation, Alzheimer’s dementia

## Abstract

**Background:**

Several medications have been applied to Alzheimer’s dementia patients (AD) but their efficacies have been insufficient. The efficacy and safety of 4 weeks of repetitive transcranial magnetic stimulation (rTMS) in Japanese AD were evaluated in this exploratory clinical trial.

**Methods:**

Forty-two patients, aged 60–93 years (average, 76.4 years), who were taking medication (> 6 months) and had Mini-Mental State Examination (MMSE) scores ≤ 25 and Clinical Dementia Rating Scale scores (CDR-J) of 1 or 2, were enrolled in this single-center, prospective, randomized, three-arm study [i.e., 120% resting motor threshold (120% RMT), 90% RMT for the bilateral dorsolateral prefrontal cortex, and Sham]. Alzheimer’s Disease Assessment Scale-Japanese Cognitive (ADAS-J cog), Montreal Cognitive Assessment (MoCA-J), Clinical Global Impression of Change (CGIC), Neuropsychiatric inventory (NPI), and EuroQOL 5 Dimensions 5-Level (EQ-5D-5L) were administered. The primary endpoint was the mean change from baseline in the MMSE score (week 4). An active rTMS session involved applying 15 trains bilaterally (40 pulses/train at 10 Hz; intertrain interval, 26 s). Participants received ≥ 8 interventions within the first 2 weeks and at least one intervention weekly in the 3rd and 4th weeks. Full Analysis set (FAS) included 40 patients [120% RMT (*n* = 15), 90% RMT (*n* = 13), and Sham (*n* = 12)].

**Results:**

In the FAS, MMSE, ADAS-J cog, MoCA-J, CDR-J, CGIC, NPI, and EQ-5D-5L scores between the three groups were not significantly different. Two patients were erroneously switched between the 120% RMT and 90% RMT groups, therefore, “as treated” patients were mainly analyzed. *Post hoc* analysis revealed significant treatment efficacy in participants with MMSE scores ≥ 15, favoring the 120% RMT group over the Sham group. Responder analysis revealed 41.7% of the 120% RMT group had a ≥ 3-point improvement in the ADAS-J cog versus 0% in the Sham group (Fisher’s exact test, *p* = 0.045). The MoCA-J showed the same tendency but was not significant. Efficacy disappeared in week 20, based on the ADAS-cog and MoCA-J. No intervention-related serious adverse events occurred.

**Conclusion:**

This paper is the first report of using rTMS in Japanese AD patients. The treatment seems safe and moderate-mild stage AD should be target population of pivotal clinical trial with 120% RMT rTMS.

## Introduction

The number of patients with Alzheimer’s dementia (AD)—a common type of dementia (Plassman et al., 2007)—is increasing globally. The number of Japanese patients with AD is also increasing because of increase of elderly population (Alz.org^®^|Japan). AD imposes a substantial financial burden on society that is predicted to worsen. However, current treatments for AD have limited efficacy. To date, only four drugs are available in Japan, and the most effective medication only improves symptoms temporarily (Mielke et al., 2012), and treatment efficacy varies among patients. Despite advances in the pharmacological treatment of AD, at present, no drug can modify the course of AD (Schneider et al., 2014). Aducanumab (Aduhelm^®^) was approved by the FDA (Food and Drug Administration) in June 2021, but is not available in Japan (Aug, 2022).

Repetitive transcranial magnetic stimulation (rTMS) effectively treats depression and is approved in Europe, the United States, and Asia (including Japan) (O’Reardon et al., 2007; George et al., 2010). Combined rTMS and cognitive training intervention has been recently reported to be effective in the treatment of patients with AD who have baseline MMSE (Mini-Mental State Examination scores) scores of 18∼26 and ADAS-J cog (Alzheimer’s disease assessment scale-Japanese cognitive) scores ≤ 30 (Sabbagh et al., 2020). High-frequency stimulation (10–20 Hz) of the bilateral (Ahmed et al., 2012; Devi et al., 2014; Rutherford et al., 2015) or left dorsolateral prefrontal cortex (DLPFC) (Cotelli et al., 2011; Haffen et al., 2012) improves general cognitive performance, episodic memory, processing speed, and language skills. On the other hand, no difference in efficacy was reported for rTMS + cognitive training versus high-frequency rTMS alone (Alcalá-Lozano et al., 2018). However, in Asia, no large clinical trials of rTMS for AD have been conducted. The skull shape of Japanese (similar to Chinese) individuals differs from those of Caucasian individuals (Ball et al., 2010). On the other hand, there is no apparent difference of resting motor threshold (RMT) between Asian and Caucasian (Suzuki et al., 2021). Thus, the efficacy of rTMS of DLPFC might differ between Caucasians and Asians.

The exact neurobiological mechanism of rTMS remains unclear, although some reports have suggested neuromodulation of neuroplasticity (Cotelli et al., 2006, 2008) and large-scale networks in the brain (Pini et al., 2018).

We developed a novel rTMS machine with Teijin Pharma Limited. The device includes a position-adjusted unit to align the TMS coil to the appropriate position, an efficient eccentric figure-8-coil (Supplementary Figure 1), and equipment for sham stimulation (Hosomi et al., 2020). The device was modified for stimulation of the bilateral DLPFC. We aimed to determine whether rTMS therapy would improve cognition in Japanese patients with AD. Patients were evaluated with the MMSE, Montreal Cognitive Assessment (MoCA-J), ADAS-J cog, Clinical Global Impression of Change (CGIC), global Clinical Dementia Rating scale (CDR-J), Neuropsychiatric Inventory (NPI), and EuroQOL 5 dimensions 5-level (EQ-5D-5L).

Stronger rTMS power would be desirable. Inclusion of 90% RMT makes us to examine the dose dependency. Therefore, we chose three arms (120% RMT, 90% RMT, sham).

## Materials and methods

### Standard protocol approvals, registrations, and patient consents

We conducted a single center, randomized, patient- and assessor-blinded, sham-controlled, parallel trial at the Osaka University Hospital (Suita, Japan). Recruitment and follow-up were conducted from April 2019 through September 2020. This study was a specified clinical trial funded by the Japan Agency for Medical Research and Development (AMED; Tokyo, Japan), and Teijin Pharma Limited offered the rTMS device. All data management, monitoring, auditing, and statistical analyses were conducted at an independent academic clinical research center of the Osaka University Hospital. Data were captured by an electric data capture (EDC) system (DATA-TRAK ONE; DataTRAK International, Inc., Mayfield Heights, OH, United States). The trial followed the guidelines of the Clinical Trials Act (Act No. 16 of April 14, 2017) and Declaration of Helsinki. The study protocol was based on our previous clinical trial (Saitoh et al., 2020), suggested by the Japanese Regulatory Authority (Pharmaceuticals and Medical Devices Agency; PMDA), and approved by Osaka University Clinical Research Review Committee (S18025). PMDA suggested that the study should include wide range of AD patients, two kinds of intervention, and long follow up periods, because this study was exploratory. In our previous study, 2 weeks of rTMS of the bilateral DLPFC resulted in insufficient improvement (Saitoh et al., 2020). Some patients with AD cannot visit hospitals by themselves, and family burden should be reduced. The patients ultimately received rTMS for at least 8 days during the first 2 weeks and at least once weekly in the third and fourth weeks. Considering that previous reports demonstrated prolonged effects after cessation of rTMS (Ahmed et al., 2012; Sabbagh et al., 2020), our protocol for the current study has a longer follow-up period (total 28 weeks) to evaluate when the effects will disappear. This trial was registered with the Japan Registry of Clinical Trials [jRCTs052180226, Scientific title: Efficacy and safety of repetitive transcranial magnetic stimulation in Alzheimer’s disease (RTMS-AD-02)].

### Patients

We enrolled patients (aged ≥ 20 years) diagnosed with probable AD according to the criteria of the National Institute of Neurological and Communicative Disorders and Stroke Alzheimer’s Disease and Related Disorders Association (NINCDS-ADRDA) (McKhann et al., 1984), who additionally met the following inclusion criteria: (1) an MMSE score of ≤ 25, (2) a baseline global Clinical Dementia Rating scale (CDR-J) score of 1 or 2, and (3) a concurrent prescription for AD medication or a history of being prescribed for > 6 months without having achieved control of cognitive function. Key exclusion criteria were severe dementia (MMSE < 10), severe mental illness, suicidal thoughts, history of seizures, being pregnant, receiving rTMS within 1 year of consenting, enrollment in other clinical trials within the 6 months before obtaining consent, and having a contraindication to rTMS (e.g., cardiac pacemaker implantation, deep brain stimulation, and metal excluding titanium in the head). Severe AD was reported to be difficult to treat with medication and rTMS, and mild AD was difficult to diagnose. We recruited participants who were outpatients of the study site and patients referred from other hospitals or clinics to this trial.

### Randomization and blinding

Patients were randomly assigned to either group [120% resting motor threshold (120% RMT) or (90% RMT)], or the Sham (group) (1:1:1). Layered replacement blocks were applied. Stratification was based on age (< 75 vs. ≥ 75 years) and MMSE score (≤ 18 vs. ≥ 19 points). Patients were allocated, using the allocation function of the EDC system. Knowledge of the treatment-group assignment was limited to those administering the intervention. Treatment-group information was stored in a lockable safe for documents and secured with a password for the EDC system. Patients and assessors were blinded, and assignments were not disclosed during the trial. To ensure blinding, no research staff switched from blinded to non-blinded or vice versa. Sham stimulation mimicked that of active stimulation (see section in “Interventions”). Dr. Eisuke Hida generated the random allocation sequence (EDC system) and EDC system assigned participants to interventions. Dr. Tomoo Mano and Dr. Youichi Saitoh enrolled participants.

### Trial schedule

**FIGURE 1:**
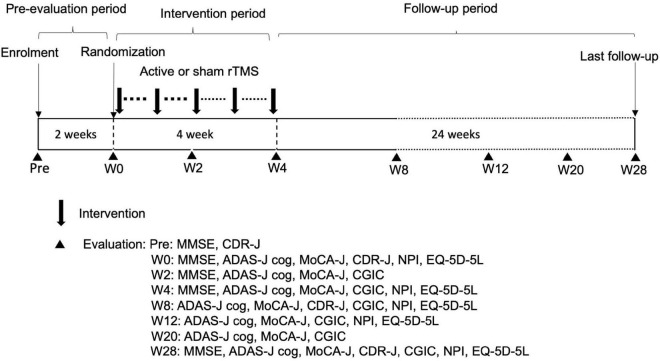
Trial schedule. W, week; rTMS, repetitive transcranial magnetic stimulation; MMSE, Mini-Mental State Examination; CDR-J, Clinical Dementia Rating scale; ADAS-J cog, Alzheimer’s Disease Assessment Scale-Japanese Cognitive; MoCA-J, Montreal Cognitive Assessment; CGIC, Clinical Global Impression of Change; NPI, Neuropsychiatric Inventory; EQ-5D-5L, EuroQOL 5 dimensions 5-level.

A geriatric physician or psychiatrist who specialized in AD assessed the eligibility of patients who provided consent. Eligible patients were randomly assigned to a treatment group. Once allocated, patients received 4-week sessions of active (120% or 90% RMT) or sham rTMS to evaluate the efficacy and safety. The patients were required to receive rTMS for at least 8 days during the first 2 weeks and at least 1 day weekly in the third and fourth weeks. All patients visited the outpatient clinic and were followed until week 28 after completing the intervention. Patients were evaluated with the MMSE, Montreal Cognitive Assessment (MoCA-J), ADAS-J cog, Clinical Global Impression of Change (CGIC), global Clinical Dementia Rating scale (CDR-J), Neuropsychiatric Inventory (NPI), and EuroQOL 5 dimensions 5-level (EQ-5D-5L). NPI can analyze behavioral and psychological symptoms of dementia (BPSD) and suggest the burden of patients’ families. The evaluation schedule is summarized in Figure 1.

The primary endpoint was the mean change from baseline in the MMSE score (week 4) because efficacy is usually prominent immediately after rTMS treatment (Saitoh et al., 2020). The secondary endpoints were the mean changes from baseline in the ADAS-J cog and MoCA-J (weeks 2, 4, 8, 12, 20, and 28) scores, evaluations of the NPI and EQ-5D-5L (weeks 4, 8, 12, and 28), and assessment of the CGIC (weeks 4, 8, 12, 20, and 28). Adverse events (AEs) were any undesirable or unintended disease condition or disorder occurring in the participant, operator, or other personnel during the use of the device. AEs were categorized as mild, moderate, or severe and coded by MedDRA/J Version 23.0. Blinded assessors collected and evaluated AEs throughout the trial period.

### Interventions

We used an rTMS system (TEN-P11; Teijin Pharma Limited) developed in collaboration with Teijin Pharma Limited (Hosomi et al., 2020). The device includes a position-adjusting unit to align TMS coils to the appropriate positions, an efficient eccentric figure-8-coil (Sekino et al., 2015; Supplementary Figure 1), and equipment for sham stimulation. The coil was similar to a conventional concentric figure-8-coil, which can reduce the driving current intensity by approximately 10% to induce a neural response that is comparable to a conventional one. The stimulation targets were sites in the bilateral DLPFC. Interventions were conducted by trained neurosurgeons, neurologists, or other medical staff under the observation of physicians.

The motor hotspot in the left hemisphere was identified by eliciting the most prominent muscle twitch in the hand. The RMT of the left motor hotspot was defined as the minimum intensity needed to induce one visible muscle twitch, which corresponded to the RMT measured using motor-evoked potentials (Hanajima et al., 2007). The left target-stimulation site was determined as 5 cm anterior to the left motor hotspot, and the right target-stimulation site was determined as the point symmetrical to the left target-stimulation site. The 5 cm anterior to the hot spot is common target for DLPFC in Japan (Kito et al., 2019). All the patients’ brains were examined by MRI before clinical trial and confirmed that there were no critical morphological problems (contusion, tumor, cortical infarction, subdural effusion, etc.). The stimulation site and RMT were determined on the first day of the intervention only. In our system, the TMS coil can be repositioned in the set position, which was determined on the first day. An active rTMS session involved 15 trains at 120% or 90% RMT to each side (40 pulses/train at 10 Hz; inter-train interval, 26 s). 120% RMT rTMS sometimes brings scalp pain in the patients. We worried about many drop out of patients because of scalp pain. Japanese Regulatory Authority (PMDA) suggested that two kinds of interventions should be included because this exploratory clinical trial may demonstrate rTMS dose dependency. The maximum rTMS intensity was 67% of the maximum stimulator output. This protocol was developed using the guidelines for the safe use of rTMS (Wassermann, 1998).

### Sham stimulation

To match the active (120% RMT and 90% RMT) and Sham stimulation methods, two pairs of electrodes were attached to the scalp near the target-stimulation sites in all groups. To generate a realistic sham stimulation (Hamada et al., 2008; Hosomi et al., 2020), electrical stimuli at 5 mA were simultaneously delivered with magnetic discharges through the sham coil, located approximately 30 cm above the scalp. Quite brief electrical square pulses (pulse width, 0.2 ms) are intermittently delivered at 10 Hz (train duration, 4 s; total number of pulses, 1,200 pulses) for the sham condition of rTMS, while constant stimulation of 5–20 min is delivered for tDCS. At least 3 min of constant tDCS is necessary to elicit cortical excitability changes (Nitsche and Paulus, 2000). rTMS-induced current densities reaching the cortical layers possibly range from 1.5 to 4.5 A/m^2^, while 2 mA-tDCS with an anode of 25 cm^2^ was shown to produce a mean current density of 0.1 A/m^2^ in the brain (Lefaucheur, 2008). Therefore, the cortical effect of the cutaneous electrical stimulation is considered to be negligible at 5 mA because of the high electrical resistance of the skull and brief duration of the stimulation.

The sham stimulation produced scalp sensations and sounds similar to active stimulation but without DLPFC stimulation. The coil position and stimulation protocol were the same as those of active stimulation.

### Sample size

Based on the assumption that efficacies would be proportional to intensity of stimulation and intervention period, we conducted a power calculation which can at least demonstrate the difference between Sham and 120% RMT groups at week 4. Estimated effect size between these two groups is based on the results from previous studies (Bentwich et al., 2011; Saitoh et al., 2020). we estimated that with 12 patients in each group, the study would have 80% power to detect absolute difference of 2.8 points (effect size of 1.14) in the primary outcome at week 4 between 120% RMT and Sham groups with the use of a two-sided *t*-test at a 0.05 significance level. We did not adjust for multiple comparisons for exploratory purpose, therefore, total of 36 patients were needed to conduct pairwise comparisons between the three groups (120% RMT, 90% RMT and Sham). Assuming withdrawals and dropouts of 10%, we decided to include 42 patients (14 patients per group). Even though the sample size determination was based on the conservative approach using *t*-test, we intended to evaluate the primary outcome based on the analysis of covariance (ANCOVA) model.

### Statistical analyses of outcomes

Continuous variables were expressed as the mean ± SD, and categorical variables were expressed as the count and percentage. Primary and secondary analyses were conducted on the full analysis set (FAS). The amount of change from the baseline was assessed using ANCOVA, with the baseline measure as a covariate in which only missing data at week 4 were imputed by carrying forward the last observation. We included interventions as fixed effects and baseline measure as a covariate in the ANCOVA model and estimated the differences in the adjusted mean change from the baseline between groups and 95% confidence intervals (CIs). We used Fisher’s exact test to compare the proportion of patients achieving more than clinically meaningful improvement (i.e., 3 points on the MMSE and ADAS-J cog; 1 point on CDR-J) at week 4 between groups. Three points on the ADAS-J cog are reasonable changes based on the clinical trial of Donepezil in Japan (Homma et al., 2000), Values of *P* < 0.05 indicated statistical significance. All analyses were performed with the use of SAS software, version 9.4 (SAS Institute, Cary, NC, United States).

## Results

**FIGURE 2:**
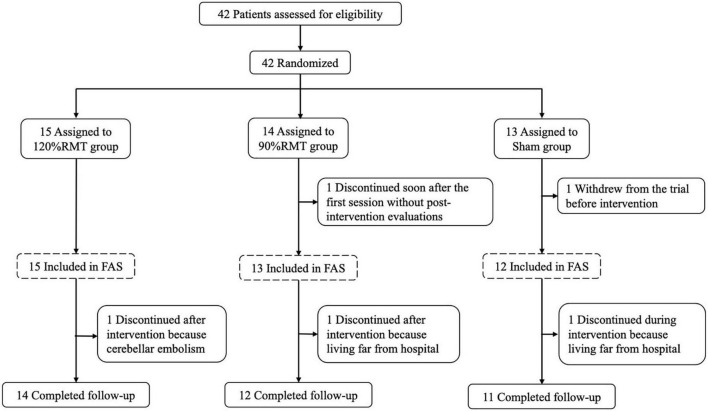
Flow diagram. RMT, resting motor threshold.

Two of 42 patients resigned to receive rTMS and examination. Forty patients (25 females and 15 males) with a mean age of 76 (SD: 7.6) years were included in the efficacy analyses of this trial. Two patients were erroneously switched between the 120% RMT and 90% RMT groups by the stimulation staff at the first intervention and received switched treatments through the study; therefore, the “As Treated” cases were summarized *post hoc* and this report mainly described “As Treated” data. Table 1 shows the patient characteristics, such as sex, age, years of education, disease duration, and the MMSE, ADAS-J cog, MoCA-J, CDR-J, NPI, and EQ-5D-5L scores. The duration of illness was 4.2 ± 2.7 years. When consent was obtained, the MMSE and ADAS-J cog scores were 18.2 ± 4.7 and 20.9 ± 6.2 respectively. Ninety-seven percent (39/40) of patients completed the intervention for up to 4 weeks, and 93% (37/40) completed the follow-up for up to 28 weeks. Compliance to the rTMS sessions is summarized in Table 2. The patients were allocated based on the MMSE score (i.e., ≤ 18 or ≥ 19 points). However, the Sham group showed better ADAS-J cog and MoCA-J scores (Table 1). The flow diagram of the study is summarized in Figure 2.

**TABLE 1:** Selected demographics of the groups.

Selected demographic	120% RMT group	90% RMT group	Sham group
Sex, n (%)			
Male	8 (53.3)	3 (23.1)	4 (33.3)
Female	7 (46.7)	10 (76.9)	8 (66.7)
Average age (y)	76.2	77.2	75.8
Education years	13.7	12.2	13.2
Average time from AD diagnosis	3.5	4.8	4.6
MMSE score	18.2 ± 4.8	17.7 ± 4.7	18.6 ± 4.9
ADAS-J cog score	21.5 ± 5.9	23.1 ± 6.3	18.0 ± 5.9
MoCA-J score	12.7 ± 5.3	13.5 ± 5.0	16.1 ± 4.4
CDR-J score	1.3 ± 0.8	1.5 ± 0.7	1.4 ± 0.7
Sum of boxes	7.1 ± 3.4	7.4 ± 3.5	6.1 ± 3.3
NPI score	11.9 ± 13.1	7.2 ± 7.9	4.8 ± 6.5
EQ-5D-5L score	0.75 ± 0.13	0.72 ± 0.13	0.80 ± 0.08

RMT, resting motor threshold; MMSE, Mini-Mental State Examination; ADAS-J cog, Alzheimer’s Disease Assessment Scale-Japanese Cognitive; MoCA-J, Montreal Cognitive Assessment; CDR-J, Clinical Dementia Rating Scale; CGIC, Clinical Global Impression of Change; NPI, Neuropsychiatric Inventory; EQ-5D-5L, EuroQOL 5 dimensions 5-level. The data are presented as the number (percent) or as the mean ± the standard deviation.

**TABLE 2:** Summary of the number of rTMS sessions.

Week	First	Second	Third	Fourth
120% RMT (*n* = 15)	4.1 ± 0.3	4.1 ± 0.3	3.1 ± 1.1	3.3 ± 1.1
90% RMT (*n* = 13)	4.0 ± 0.0	4.0 ± 0.0	3.2 ± 1.1	2.9 ± 1.3
Sham (*n* = 12)	4.2 ± 0.4	3.9 ± 0.5	2.2 ± 1.3	2.3 ± 1.3

The data are presented as the mean ± the standard deviation. rTMS, repetitive transcranial magnetic stimulation.

### Safety

In total, 19 patients reported AEs related to the intervention (7, 7, and 4 in the 120% RMT, 90% RMT, and Sham groups). The AEs were confirmed by the physician in charge of an independent medical review committee. Most AEs were anticipated events that are commonly associated with rTMS—especially pain of skin (i.e., scalp) (46%). Serious AEs were also detected: one case each of cerebellar embolism, cholecystitis, atrioventricular block, loss of consciousness, and lumber spinal stenosis. These serious AEs were observed during the follow-up periods. The blind physician determined that the serious AEs were unrelated to the study procedure or device (Table 3). In this study, no patient has developed seizures after rTMS.

**TABLE 3:** Adverse events.

	120% RMT group (*N* = 15)	90% RMT group (*N* = 14)	Sham group (*N* = 12)
Pain of skin	7 (46.7)	7 (50)	4 (33)
Muscle contractions involuntary	1 (6.7)		
Toothache	1 (6.7)		
Pain in jaw	1 (6.7)		
Fall	3 (20)		1 (8.3)
Herpes zoster			1 (8.3)
Conjunctival hemorrhage		1 (7.1)	
Constipation		1 (7.1)	
Ligament Sprain	1 (6.7)		
Hemorrhage Subcutaneous	1 (6.7)		
Internal hemorrhage	1 (6.7)		
Embolic stroke	1 (6.7)		
Cholecystitis		1 (7.1)	
Atrioventricular block	1 (6.7)		
Loss of consciousness			1 (8.3)
Lumbar spinal stenosis	1 (6.7)		
Abdominal discomfort		1 (7.1)	
Pollakiuria	1 (6.7)		
Pain in extremity	1 (6.7)		
Back pain	1 (6.7)		
Groin pain	1 (6.7)		

Data are presented as n (%) and are corrected from the safety analysis set (SAS). Some participants complain of multiple adverse events. Adverse events were coded, using MedDRA/J version 23.0. “Pain of skin,” “muscle contractions involuntary,” “toothache” and “pain in jaw” are considered to be related with interventions by the physician in charge of an independent medical review committee. RMT, resting motor threshold.

### Efficacy

#### Efficacy in full analysis set and as treated groups

In the FAS, there were no significant differences between the 120% RMT, 90% RMT, and Sham groups in the MMSE, ADAS-J cog, MoCA-J, CDR-J, CGIC, NPI, and EQ-5D-5L scores.

In As Treated group, there were no significant between-group differences for all outcomes in this case either (Table 4). With respect to the MMSE and MoCA-J scores, adjusted mean changes from baseline at week 4 were 1.5 (95% CI, 0.4 to 2.7) in the 120% RMT group, −0.7 (95% CI, −1.9 to 0.5) in the 90% RMT group, 0.5 (95% CI, −0.8 to 1.8) in the Sham group, and 1.5 (95% CI, 0.1 to 2.9) in the 120% RMT group, 0.7 (95% CI, −0.8 to 2.2) in the 90% RMT group, 1.3 (95% CI, −0.3 to 2.9) in the Sham group, respectively. Further, regarding the ADAS-J cog score, adjusted mean change from baseline at week 4 was −1.3 (95% CI, −3.2 to 0.6) in the 120% RMT group, −1.0 (95% CI, −3.1 to 1.1) in the 90% RMT group, and −0.3 (95% CI, −2.6 to 1.9) in the Sham group.

**TABLE 4:** Efficacy outcomes in as treated.

Adjusted mean change from baseline (95% CI) at week 4	Sham		90% RMT		120% RMT		Adjusted mean difference between groups (95% CI)			
							90% RMT vs. Sham	*P*-value^†^	120% RMT vs. Sham	*P*-value^†^
	N		N		N					
MMSE	12	0.5 (−0.8, 1.8)	13	−0.7 (−1.9, 0.5)	15	1.5 (0.4, 2.7)	−1.2 (−3.0, 0.5)	0.17	1.0 (−0.7, 2.7)	0.23
ADAS-J cog	12	−0.3 (−2.6, 1.9)	13	−1.0 (−3.1, 1.1)	15	−1.3 (−3.2, 0.6)	−0.7 (−3.8, 2.5)	0.66	−1.0 (−4.0, 2.0)	0.50
MoCA-J	12	1.3 (−0.3, 2.9)	13	0.7 (−0.8, 2.2)	15	1.5 (0.1, 2.9)	−0.6 (−2.8, 1.6)	0.60	0.2 (−2.0, 2.3)	0.88
NPI	12	−4.4 (−8.4, −0.4)	13	−0.1 (−3.9, 3.6)	15	−2.2 (−5.8, 1.4)	4.3 (−1.2, 9.7)	0.12	2.2 (−3.3, 7.7)	0.41
EQ-5D-5L	12	0.06 (0.01, 0.12)	13	0.02 (−0.02, 0.07)	15	0.04 (0.00, 0.08)	−0.04 (−0.11, 0.03)	0.26	−0.03 (−0.01, 0.04)	0.43

**Proportion of patients** **with improvement^§^ at week 4**		**Sham**		**90% RMT**		**120% RMT**		**90% RMT vs. Sham**		**120% RMT vs. Sham**
								***P*-value^‡^**		***P*-value^‡^**

MMSE		3/12 (25.0%)		1/13 (7.7%)		4/15 (26.7%)		0.32		1.00
ADAS-J cog		2/12 (16.7%)		4/13 (30.8%)		5/15 (33.3%)		0.64		0.41
CDR-J		3/12 (25.0%)		2/13 (15.4%)		2/15 (13.3%)		0.64		0.63
CGIC		1/12 (8.3%)		0/13 (0.0%)		1/15 (6.7%)		0.48		1.00

^†^The *P* value was calculated with the use of analysis of covariance (ANCOVA) with adjustment for baseline score as a covariate.

^‡^The *P* value was calculated with the use of Fisher’s exact test.

^§^Improvement is defined as 3 points change in MMSE, ADAS-J cog, and 1 point change in CDR-J.

The responder analysis of the ADAS-J cog score (≥ 3 points of ADAS-J cog) showed 5 of 15 (33.3%) in the 120% RMT group, 4 of 13 (30.8%) in the 90% RMT group, and 2 of 12 (16.7%) in the Sham group at week 4 (Fisher’s exact test, *P* = 0.41 between the 120% RMT and Sham groups, *P* = 0.64 between the 90% RMT and Sham groups) (Table 4).

#### Efficacy in as treated group (MMSE ≥ 15)

Severe dementia patients (ADAS-cog > 30) were reported not to be good candidate for rTMS treatments (Sabbagh et al., 2020); therefore, we analyzed patients whose MMSE scores were ≥ 15 (120% RMT group: 12, 90% RMT group: 10, and Sham group: 9 cases) in the within- and between-groups. In the between-group analysis of change from baseline at week 4, the MMSE, ADAS-J cog, and MoCA-J scores did not reach statistical significance. However, the 120% RMT group showed a tendency of improvement at week 4. Furthermore, responder analysis of the ADAS-J cog score (≥ 3 points of ADAS-J cog) showed 5 of 12 (41.7%) in the 120% RMT group, 3 of 10 (30%) in the 90% RMT group, and 0 of 9 (0%) in the Sham group at week 4, and there was a significant difference between the 120% RMT and Sham groups (Fisher’s exact test *P* = 0.045).

#### The duration of efficacy and compliance

**FIGURE 3:**
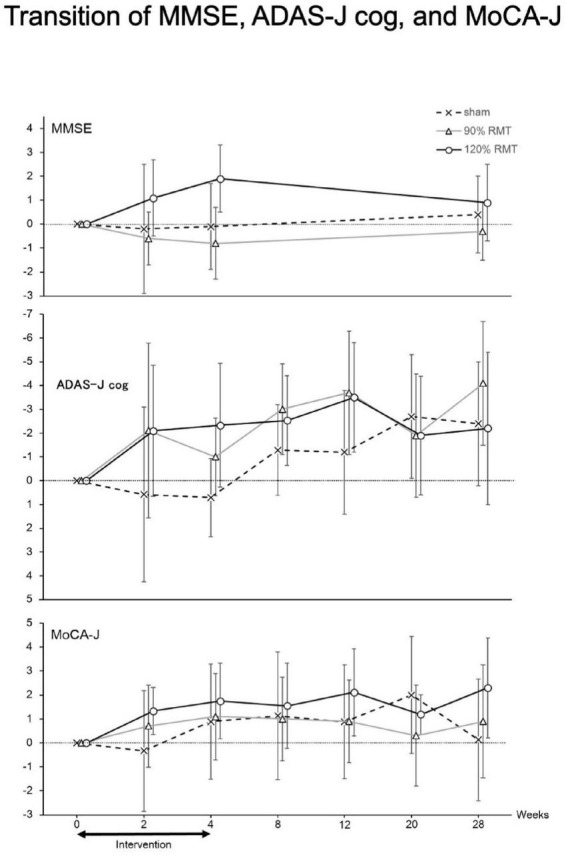
Transition of MMSE, ADAS-J cog and MoCA-J. Thus, we only analyzed patients with MMSE scores > 15 and the resulting groupings were as follows: 120% RMT group, 12 patients; 90% RMT group, 10 patients; and Sham group, 9 patients. Among the patients, the 120% RMT and 90% RMT groups showed several improvements in the MMSE, ADAS-J cog and MoCA-J scores. Treatment efficacy appeared to disappear at 20 weeks. MMSE, Mini-Mental State Examination, ADAS-J cog, Alzheimer’s Disease Assessment Scale-Japanese Cognitive, MoCA-J, Montreal Cognitive Assessment, RMT, resting motor threshold, vertical axis: improved points from the baseline.

The efficacy appeared to disappear at week 20 (Figure 3). The MMSE, ADAS-J cog, and MoCA-J scores showed similar tendencies. In this study, the patients received intervention at least once a week during the third and fourth weeks. Practically, most of the patients received rTMS for 3 days in the third and fourth weeks. The number of rTMS sessions for all weeks in all groups is summarized in Table 2. The number of rTMS sessions was not significantly different between the groups.

## Discussion

The efficacy and safety of 4 weeks of rTMS in Japanese patients with AD were evaluated in this exploratory clinical trial. Reports on the use of rTMS in Japanese patients with AD appear to be rare. In the FAS analysis, there were no significant efficacies. In the *post hoc* analysis, we found that treatment efficacy was significant in participants with an MMSE score of ≥ 15 (in favor of the 120% RMT group over the Sham group) and that no intervention-related serious AEs occurred. The efficacy appears to disappear at 20 weeks based on the ADAS-J cog and MoCA-J scores (Figure 3). We showed that the efficacy of rTMS continues after 4 weeks of intervention.

The major reason of negative data in FAS was due to inclusion of wide range of AD patients (severe mild). The second reason was small sample size. The Japanese Regulatory Authority (PMDA) suggested that this exploratory clinical trial should include wide range of AD and two kinds of intervention.

In previous reports (Ahmed et al., 2012; Sabbagh et al., 2020), patients with severe AD were poor candidates for rTMS, and the efficacy was prolonged or emphasized after rTMS treatment. It has been reported that high frequency rTMS of the bilateral DLPFC for 5 consecutive days improved cognition for 3 months in mild and moderate AD (Ahmed et al., 2012). In our study, high frequency rTMS of the bilateral DLPFC for 4 weeks appeared to be also effective at weeks 12 and 20.

The mechanism of action of rTMS in AD is unclear. A possibility is that the dopamine system between the prefrontal cortex and basal ganglia is activated by DLPFC stimulation, similar to depression treatment (Sawaguchi et al., 1990; Ott and Nieder, 2019). Depressive symptoms are common in AD and occur in approximately 40% of patients with AD (Weiner et al., 1994). Depressive mood improved in some patients in our study (based on family members’ report)—most of whom belonged to the 120% RMT group. However, in one study, the left DLPFC was stimulated in depressed patients who then performed a switching task that required controlling attention between visual and auditory cues. It was found that patients who received active stimulation had improved reaction times, whereas those who received sham stimulation showed no improvement (Vanderhasselt et al., 2006). In other report (Suarez-Moreno et al., 2022), long-term improvement in apathy and more general cognitive improvement only in patients who responded well to the initial 6-week NeuroAD™ treatment.

It is much likely that the stimulation of DLPFC might have restored the rich cholinergic innervation that these are receive from the basal forebrain, that are known to be impaired also in mild cognitive impairment (MCI) patients (Martorana et al., 2014) and that is important also in healthy people for memory tasks, as demonstrated in a TMS study (Bonni et al., 2017).

Another possibility is a theory that involves induction of long-term potentiation (LTP) and the modulation of brain plasticity (Sabbagh et al., 2020). Cognitive improvement with rTMS has been associated to the improvement of LTP mechanisms (Li et al., 2021). LTP impairment has been proved to be associated to prediction of cognitive impairment (Di Lorenzo et al., 2020) and CSF Tau levels (Koch et al., 2011). Moreover, the plasticity of connections arising from DLPFC have been investigated (Di Lorenzo et al., 2018) in AD patients and modulating their plasticity with a rTMS treatment could restore this impaired pathway.

In severe AD, the dopaminergic and cholinergic systems and brain plasticity are irreversibly damaged (Alagona et al., 2001; Ferreri et al., 2003; Ahmed et al., 2012; Martorana et al., 2014). The motor threshold decreases and is correlated with AD progression. Considering this, clinicians should target patients with mild to moderate AD, and the motor threshold may indicate the appropriate candidates (Sabbagh et al., 2020).

Despite hundreds of clinical trials of medications, only some medications are available. Non-drug treatments are receiving increased attention, and rTMS is a representative non-drug neuromodulatory treatment. Regarding other non-drug treatments, it has been shown that aerobic exercise increases oxygen uptake and improves cognition and neuropsychiatric symptoms in patients (Sobol et al., 2018). Several studies have reported positive results on cognitive function by combining rTMS with specific behavioral or cognitive interventions (Zhao et al., 2017; Sabbagh et al., 2020). However, the NeuroAD™ system, which delivers neuronavigated focal rTMS concurrently with cognitive training exercises, failed in a pivotal clinical trial. This failure may have resulted from the inclusion of too many patients with severe AD (ADAS-J cog ≤ 30) in the trial. It has been reported that patients with severe AD (ADAS-J cog > 30) are not good candidates for NeuroAD™ treatment (Sabbagh et al., 2020).

A score of 30 on the ADAS-cog is equivalent to a 17 on the MMSE (Ito et al., 2010). We hypothesized that a baseline MMSE score of approximately 17 could be a cutoff score for effective intervention. For assessing the clinical cutoff validity, we plotted scatter plots to display the relationship between the MMSE scores at baseline and change from baseline scores in the ADAS-J cog at week 4 (Supplementary Figure 2). Curves were fitted with a locally weighted scatter plot smoother function with borders indicating 95% CI. Our analysis showed that rTMS tends to be effective for AD—especially among patients in the 120% RMT group whose MMSE baseline scores were ≥ 15. Therefore, in the current study, we further analyzed the patients with AD (MMSE ≥ 15).

The patients in the 120% RMT group typically complained of scalp pain during rTMS; however, after a few days of intervention, they were able to endure it. In this study, no patients in the 120% RMT group missed the intervention. The frontal lobes of the patients were usually atrophic; therefore, 120% RMT was preferable to 90% RMT. The motor threshold was weakly correlated with the baseline MMSE score (*r* = −0.31). A previous report found that the motor threshold was significantly correlated with the baseline ADAS-J cog scores (*r* = −0.4) (Sabbagh et al., 2020). There are similar correlation tendencies between motor threshold and cognitive function.

## Limitations

In this study, two patients were erroneously switched between the 120% RMT and 90% RMT groups, therefore, the “As treated” cases were summarized. Other limitation of this study was that it was an exploratory study. Therefore, each group had a small sample size and the optimal number of rTMS sessions at weeks 3 and 4 could not be determined. Another limitation is the lack of pathophysiological biomarkers of AD (Dubois et al., 2014).

## Conclusion

Reports of rTMS for Japanese patients with AD appear to be rare. The results appear safe and moderate-mild stage AD should be target population of pivotal clinical trial with 120% RMT rTMS. Pivotal clinical trials are needed to obtain the approval of the Japanese government. Furthermore, the assessment of patients with AD for longer durations is needed for application to clinical practice.

## Data availability statement

The original contributions presented in this study are included in the article/Supplementary material, further inquiries can be directed to the corresponding author.

## Ethics statement

The studies involving human participants were reviewed and approved by Osaka University Clinical Research Review Committee (CRB5180007). The patients/participants provided their written informed consent to participate in this study.

## Author contributions

YS and TM designed and conducted the study, including enrollment of patients, and the interpretation of data. YS drafted the manuscript and had final responsibility for submission of the manuscript. ST, KH, and YT contributed to the interpretation of data. NM, AMa, and YJ performed rTMS treatments. YT determined the adverse events. TY and HH managed the project. TY and AMi developed the statistical analysis plan and were responsible for statistical analysis and contributed the interpretation of data. All authors contributed to the article and approved the submitted version.

## Supplementary material

Supplementary Figure 1 **Left**: The schema of eccentric coil. Spherical shape is ergonomically considered. **Right**: In the clinical trial model, two coils were equipped.

Supplementary Figure 2 LOESS curve smooth parameter = 0.6. To assess the clinical cutoff validity, we plotted scatter plots to display the relationship between MMSE baseline scores and the change from baseline in the ADAS-cog scores at week 4. Curves were fitted with the locally weighted scatter plot smoother (LOESS) function, with borders indicating 95% CI. MMSE, Mini-Mental State Examination; ADAS-cog, Alzheimer’s Disease Assessment Scale-Cognitive; RMT, resting motor threshold; CI, confidence interval.
