# Supplementary figures and images for: Randomized, sham-controlled, clinical trial of repetitive transcranial magnetic stimulation for patients with Alzheimer’s dementia in Japan
Source: Front Aging Neurosci. 2022 Oct 13;14:993306. doi: 10.3389/fnagi.2022.993306 (PMC9606646; doi:10.3389/fnagi.2022.993306)

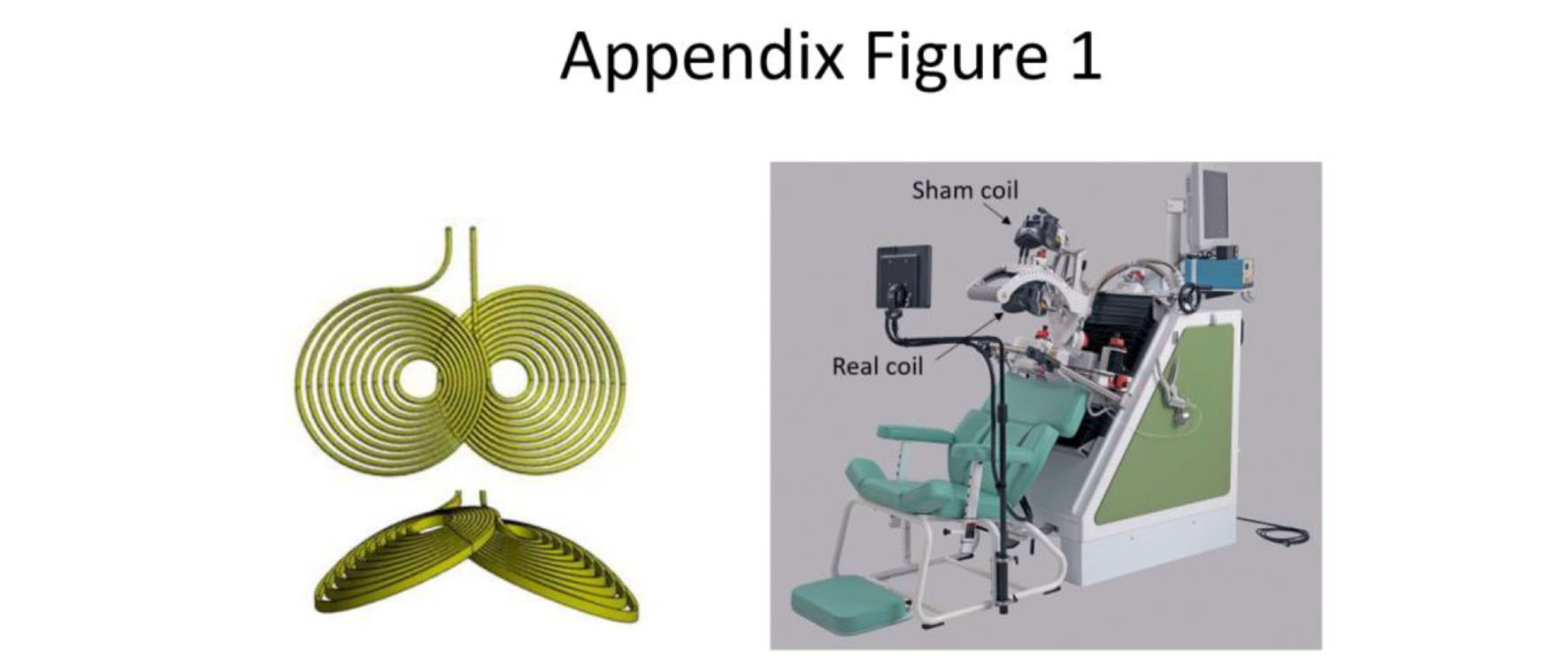

Supplement: Supplementary Figure 1 — Left: The schema of eccentric coil. Spherical shape is ergonomically considered. Right: In the clinical trial model, two coils were equipped. [file Image_1.JPEG]

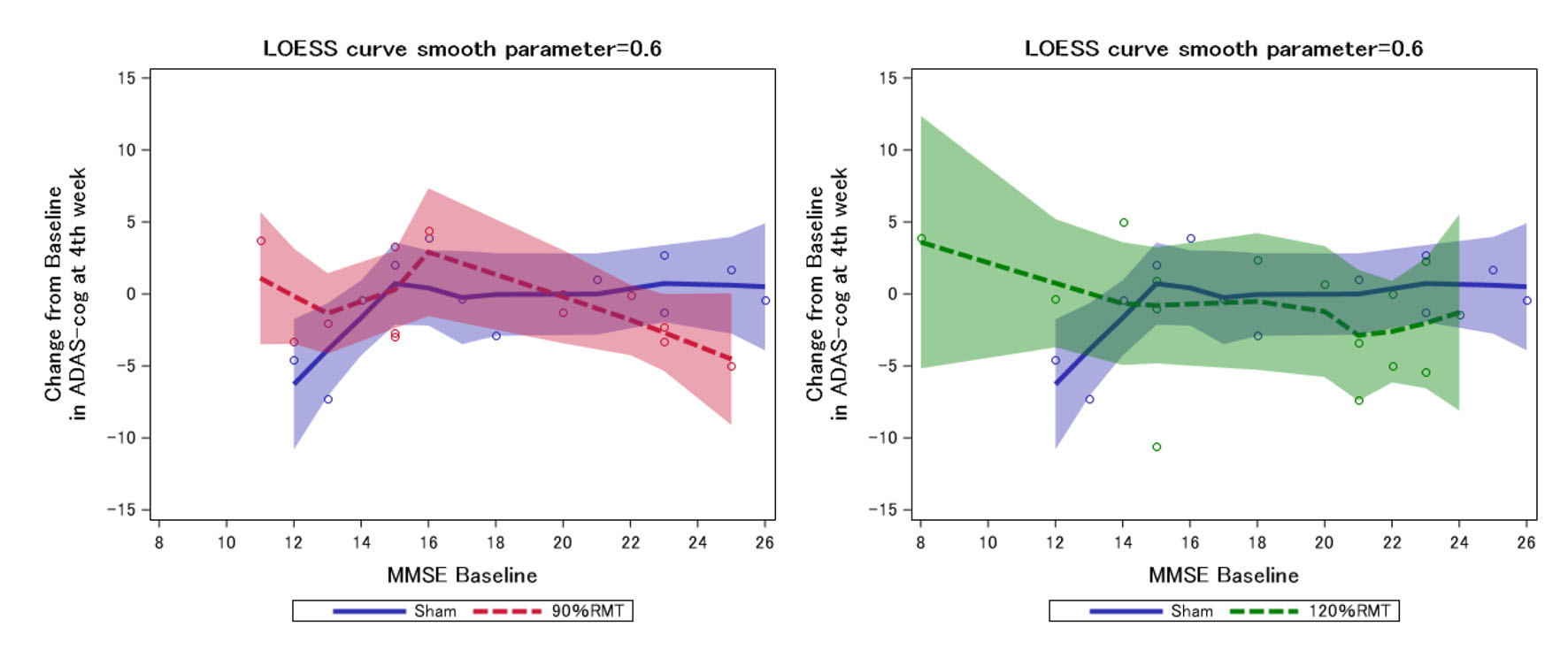

Supplement: Supplementary Figure 2 — LOESS curve smooth parameter = 0.6. To assess the clinical cutoff validity, we plotted scatter plots to display the relationship between MMSE baseline scores and the change from baseline in the ADAS-cog scores at week 4. Curves were fitted with the locally weighted scatter plot smoother (LOESS) function, with borders indicating 95% CI. MMSE, Mini-Mental State Examination; ADAS-cog, Alzheimer’s Disease Assessment Scale-Cognitive; RMT, resting motor threshold; CI, confidence interval. [file Image_2.JPEG]
